# Allen County, Indiana, Medical Association

**Published:** 1867-07

**Authors:** 


### ALLEN COUNTY, INDIANA, MEDICAL ASSOCIATION.

Pursuant to previous announcement, the Allen County Medical Society met at 3 o'clock P.M., on Thursday, June 6th, 1867, in the reading room of the Young Men's Christian Association, for its annual session, together with a large number of invited guests—members of the profession—from several of our neighboring Counties, Wabash, Huntington, Columbia City, Auburn, and Buffton, Ind., and Van Wert, Hicksville, Defiance, and Antwerp, Ohio, were amongst the places outside this County, represented in the meeting.

The meeting was called to order by the President elect, Dr. T. C. Eakin, of Monroeville, and Dr. J. S. Gregg, of Fort Wayne, previously elected for the ensuing year, took his seat as Secretary.

The Chair then appointed as the Committee of Censors provided for by the Constitution, Drs. Woodworth, Erwin, and H. P. Ayres.

The retiring Secretary, Dr. A. J. Erwin, and Treasurer, Dr. W. H. Brooks, presented their annual reports, which were adopted.

An invitation was then given to visiting members of the profession, to become permanent members of this Society, when the following gentlemen, by their consent, were duly proposed and elected for membership, and were admitted by signing the Constitution: T. Davenport, Warsaw, Ind.; D. G. Linvill, Columbia City, Ind.; F. McCoy, Columbia City, Ind.; W. T. Ferguson, Columbia City, Ind.; J. M. Josse, Fort Wayne; Charles Meyer, Fort Wayne; A. Engle, Monroeville; James McCleerey, Bluffton; Joseph Emanuel, Spencerville; C. S. Melsheimer, Bluffton; W. R. Winton, Wabash; A. D. Emanuel, Antwerp, Ohio; A. McDaniel, Antwerp, Ohio; L. G. Thacker, Defiance, Ohio; J. B. Cassbeer, Auburn, Ind.; Jacob W. Abel, Leslie, Ohio; B. F. Cessna, Van Wert, Ohio; Wm. Longsworth, Van Wert, Ohio; D. W. Champer, Bluffton, Ind.; Wm. Dugal, New Haven, Ind.

A motion was then adopted making all other invited guests members of the Society.

The following resolution was then offered by Dr. H. P. Ayres:

*Resolved*, That the principles which govern us in the treatment of disease generally are the fundamental principles of correct medication of Asiatic cholera.

Prof. J. Adams Allen, of the Rush Medical College, by invitation, then came forward and addressed the Society at some length, on the subject of the resolution, giving an interesting history of the cholera epidemic in Chicago, last summer and fall. He dwelt with much emphasis on the local causes which increased the malignancy of the disease in that city, amongst which were the filthy condition, in certain parts of the city, of the alleys and streets with rotten wood pavements, decaying organic mattars in cellars, imperfect drainage, bad condition of sewers, the want of proper ventilation of many houses, from cellar to attic, etc.

He urged the necessisy of the practical application of the principle that "an ounce of prevention is worth a pound of cure;" and that cholera is much more easily prevented by a rigid enforcement of correct sanitary measures than cured. He showed clearly that when the scourge could not be entirely prevented by such regulations, its malignance could be very greatly mitigated, and rendered much more manageable by a proper course of medication.

On its treatment we have no *specifics*. Each case must be treated upon general principles, as expressed in the resolution, the best treatment being modified to suit the modifications in each particular case.

After Prof. Allen resumed his seat, the discussion was continued for some time, by Dr. Woodworth, who called special attention to the use of disinfectants, in the prevention of disease, and Dr. Winton, and others.

After some further preliminaries, uninteresting to the public, the Society adjourned, to meet in Hamilton's Hall, at 8 o'clock, to hear the public lecture by Prof. Allen, on "The True Basis of Medical Science."

The discourse was a highly philosophical one—well calculated to show the many fallacies of the reasonings of many half-learned doctors, as well as charlatans in all professions, especially that very common false mode of reasoning, called the *post hoc ergo propter hoc* method. The lecture abounded with touches of quiet humor and graces of literature, which showed the speaker was an accomplished scholar, and a highly cultivated gentleman. The subject of the discourse was, it is true, not so well calculated to amuse a promiscuous audience as to instruct a literary one; but considering the *personnel* of his auditors, it was well chosen, and, as we have said before, highly appreciated.

After the close of the lecture, the members of the Society, with invited guests, to the number of nearly one hundred, adjourned to the spacious dining hall of the Aveline House, where a sumptuous festival had been prepared for the occasion, in Messrs. Miller & Moritz's best style. As the eatables and drinkables went in, wit and humor began to flow out.

There will be a stated meeting of the Society on the first Tuesday of each month. F. W. D.

